# *trans*-Tetra­aqua­bis(nicotinamide-κ*N*)cadmium(II) biphenyl-4,4′-disulfonate

**DOI:** 10.1107/S1600536808002390

**Published:** 2008-01-25

**Authors:** Chunyuan Li, Min Chen, Changlun Shao

**Affiliations:** a Department of Applied Chemistry, College of Science, South China Agricultural University, Guangzhou 510642, People’s Republic of China; b Centre of Experimental Teaching of Common Basic Courses, South China Agricultural University, Guangzhou 510642, People’s Republic of China; c School of Medicine and Pharmacy, Ocean University of China, Qingdao 266003, People’s Republic of China

## Abstract

In the title compound, [Cd(C_6_H_6_N_2_O)_2_(H_2_O)_4_](C_10_H_8_O_6_S_2_), the Cd^II^ ion is located on a crystallographic inversion centre. An octa­hedral coordination geometry is defined by four water mol­ecules in one plane, and two *trans* N-atom donors of the nicotinamide ligands. The biphenyl-4,4′-disulfonate anion also lies on a crystallographic inversion centre. In the crystal structure, the complex cations are connected to the counter-anions *via* N—H⋯O and O—H⋯O hydrogen bonds, forming a three-dimensional network.

## Related literature

For related literature, see: Beatty (2001 ▶); Christer *et al.* (2004 ▶); Holman *et al.* (2001 ▶); Lian & Li (2007*a* ▶,*b* ▶,*c* ▶,*d* ▶).
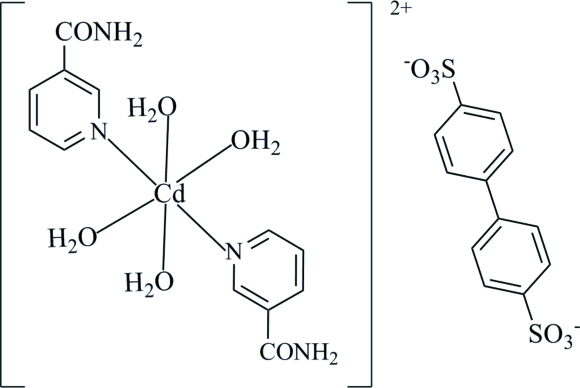


## Experimental

### 

#### Crystal data

[Cd(C_6_H_6_N_2_O)_2_(H_2_O)_4_](C_10_H_8_O_6_S_2_)
*M* *_r_* = 741.02
Monoclinic, 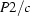

*a* = 14.742 (8) Å
*b* = 6.899 (4) Å
*c* = 15.292 (8) Å
β = 110.980 (9)°
*V* = 1452.2 (13) Å^3^
*Z* = 2
Mo *K*α radiation
μ = 0.96 mm^−1^
*T* = 298 (2) K
0.40 × 0.36 × 0.31 mm

#### Data collection

Bruker SMART APEX CCD diffractometer
Absorption correction: multi-scan (*SADABS*; Sheldrick, 1996 ▶) *T*_min_ = 0.682, *T*_max_ = 0.741
7656 measured reflections
2842 independent reflections
2477 reflections with *I* > 2σ(*I*)
*R*_int_ = 0.034

#### Refinement

*R*[*F* ^2^ > 2σ(*F* ^2^)] = 0.030
*wR*(*F* ^2^) = 0.092
*S* = 1.07
2842 reflections
197 parameters
H-atom parameters constrained
Δρ_max_ = 0.57 e Å^−3^
Δρ_min_ = −1.19 e Å^−3^

### 

Data collection: *SMART* (Bruker, 1997 ▶); cell refinement: *SAINT* (Bruker, 1997 ▶); data reduction: *SAINT*; program(s) used to solve structure: *SHELXS97* (Sheldrick, 2008 ▶); program(s) used to refine structure: *SHELXL97* (Sheldrick, 2008 ▶); molecular graphics: *SHELXTL* (Sheldrick, 2008 ▶); software used to prepare material for publication: *SHELXTL*.

## Supplementary Material

Crystal structure: contains datablocks global, I. DOI: 10.1107/S1600536808002390/fj2097sup1.cif

Structure factors: contains datablocks I. DOI: 10.1107/S1600536808002390/fj2097Isup2.hkl

Additional supplementary materials:  crystallographic information; 3D view; checkCIF report

## Figures and Tables

**Table 1:** Hydrogen-bond geometry (Å, °)

*D*—H⋯*A*	*D*—H	H⋯*A*	*D*⋯*A*	*D*—H⋯*A*
O5—H5*A*⋯O3^i^	0.81	2.03	2.832 (3)	171
O5—H5*B*⋯O4^ii^	0.86	1.83	2.676 (2)	172
O6—H6*A*⋯O3	0.85	1.93	2.777 (3)	178
O6—H6*B*⋯O2^iii^	0.83	1.89	2.716 (3)	171
N2—H2*A*⋯O1^iv^	0.86	2.08	2.932 (2)	171
N2—H2*B*⋯O2^v^	0.86	2.17	3.025 (3)	172

Symmetry codes: (i) 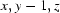; (ii) 
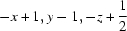
; (iii) 
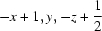
; (iv) 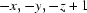; (v) 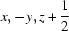.

**Fig. 1.**
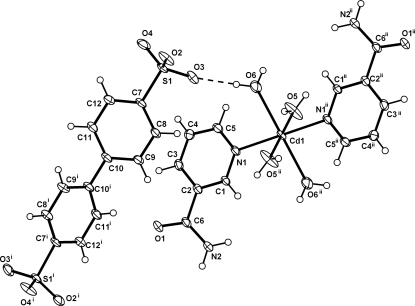
The molecular structure of (1), with the atom labelling scheme. Displacement ellipsoids are drawn at the 40% probability level. H atoms are represented as spheres of arbitrary radii. Hydrogen bonds are shown as dashed lines. Symmetry codes:(i) -x, y, -z + 1/2; (ii): -x + 1, -y, -z + 1.

**Fig. 2.**
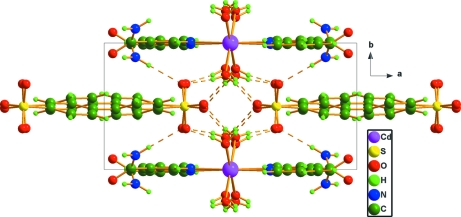
The three dimensional H-bonded network viewed from b axis direction. Hydrogen bonds are shown as dashed lines.

## supplementary crystallographic information

### Comment

The strong and directional nature of hydrogen bonds is exploited in the organized self-assembly of molecules in the solid state. Amides are commonly used functional groups in crystal engineering owing to the inherent coordination and hydrogen bonding donor/acceptor functionalities, and were used to construct extended frameworks sustained both by hydrogen bonds and coordination bonds (Beatty 2001; Christer *et al.*, 2004). On the other hand, the sulfonate group is a suitable hydrogen-bond acceptor, and has been used to build extended frameworks. (Holman *et al.*, 2001; Lian & Li, 2007*a*,b,c,d) In this paper, we report the synthesis and crystal structure of the title compound.

In the title compound, the metal centre, located on a crystallographic inversion centre, is in an octahedral geometry defined by four O atoms from four aqua ligands,respectively, and two *trans*-positioned N-atom donors of the nicotinamide ligands, as shown in Fig 1. The amide substituents are twisted away from the conformation in the pyridine ring plane by 37.6 (6)°. The biphenyl-4,4'-disulfonate anion also lies on a crystallographic inversion centre. The planes through phenyl ring in biphenyl-4,4'-disulfonate are twisted by 28.5 (7)°. In the crystal structure, all the amide groups are involved in amide-amide hydrogen bonded linkage in head to head fashion leading to infinite chains of the cations. These chains are linked by hydrogen bonds formed by the remaining N—H protons on the nicotinamide and sulfonate oxygen atoms, and coordinated water molecules and sulfonate oxygen atoms into three-dimensional networks as shown in Fig 2.

### Experimental

Nicotinamide (0.050 g, 0.4 mmol) was added with constant stirring to an aqueous solution (10 mL) of Cd(CH_3_COO)_2_.2H_2_O (0.058 g, 0.2 mmol). The solution was then treated with disodium biphenyl-4,4'-disulfonate (0.070 g, 0.2 mmol). Colourless crystals of the title complex were collected by slow evaporation at room temperature after 7 days, (80% yield based on Cd).

### Refinement

All the non-H atoms were refined with anisotropic thermal parameters.

H atoms attached to C or N atoms were placed in geometrically calculated positions (C—H = 0.93 Å, N–H=0.86 Å), and refined as riding with *U*_iso_(H) = 1.2*U*_eq_(C or N).

The O-bound H atoms were located in difference maps and refined as riding on the attached O atoms, with *U*_iso_(H) = 1.2*U*_eq_(O).

### Crystal data

**Table:**

[Cd(C_6_H_6_N_2_O)_2_(H_2_O)_4_](C_10_H_8_O_6_S_2_)	*F*_000_ = 752
*M**_r_* = 741.02	*D*_x_ = 1.695 Mg m^−^^3^
Monoclinic, *P*2/*c*	Mo *K*α radiation λ = 0.71073 Å
Hall symbol: -P 2yc	Cell parameters from 7656 reflections
*a* = 14.742 (8) Å	θ = 12–18º
*b* = 6.899 (4) Å	µ = 0.97 mm^−^^1^
*c* = 15.292 (8) Å	*T* = 298 (2) K
β = 110.980 (9)º	Block, colourless
*V* = 1452.2 (13) Å^3^	0.40 × 0.36 × 0.31 mm
*Z* = 2	

### Data collection

**Table:**

Bruker SMART APEX CCD diffractometer	2842 independent reflections
Radiation source: fine-focus sealed tube	2477 reflections with *I* > 2σ(*I*)
Monochromator: graphite	*R*_int_ = 0.034
*T* = 298(2) K	θ_max_ = 26.0º
φ and ω scans	θ_min_ = 3.3º
Absorption correction: multi-scan(SADABS; Sheldrick, 1996)	*h* = −18→12
*T*_min_ = 0.682, *T*_max_ = 0.741	*k* = −8→7
7656 measured reflections	*l* = −13→18

### Refinement

**Table:**

Refinement on *F*^2^	Hydrogen site location: inferred from neighbouring sites
Least-squares matrix: full	H-atom parameters constrained
*R*[*F*^2^ > 2σ(*F*^2^)] = 0.030	*w* = 1/[σ^2^(*F*_o_^2^) + (0.0578*P*)^2^ + 0.3728*P*] where *P* = (*F*_o_^2^ + 2*F*_c_^2^)/3
*wR*(*F*^2^) = 0.092	(Δ/σ)_max_ < 0.001
*S* = 1.07	Δρ_max_ = 0.58 e Å^−^^3^
2842 reflections	Δρ_min_ = −1.19 e Å^−^^3^
197 parameters	Extinction correction: SHELXL97 (Sheldrick, 2008), Fc^*^=kFc[1+0.001xFc^2^λ^3^/sin(2θ)]^-1/4^
Primary atom site location: structure-invariant direct methods	Extinction coefficient: 0.052 (2)
Secondary atom site location: difference Fourier map	

### Special details

**Table:**

Geometry. All e.s.d.'s (except the e.s.d. in the dihedral angle between two l.s. planes) are estimated using the full covariance matrix. The cell e.s.d.'s are taken into account individually in the estimation of e.s.d.'s in distances, angles and torsion angles; correlations between e.s.d.'s in cell parameters are only used when they are defined by crystal symmetry. An approximate (isotropic) treatment of cell e.s.d.'s is used for estimating e.s.d.'s involving l.s. planes.
Refinement. Refinement of *F*^2^ against ALL reflections. The weighted *R*-factor *wR* and goodness of fit *S* are based on *F*^2^, conventional *R*-factors *R* are based on *F*, with *F* set to zero for negative *F*^2^. The threshold expression of *F*^2^ > σ(*F*^2^) is used only for calculating *R*-factors(gt) *etc*. and is not relevant to the choice of reflections for refinement. *R*-factors based on *F*^2^ are statistically about twice as large as those based on *F*, and *R*- factors based on ALL data will be even larger.

### Fractional atomic coordinates and isotropic or equivalent isotropic displacement parameters (Å^2^)

**Table:**

	*x*	*y*	*z*	*U*_iso_*/*U*_eq_	
Cd1	0.5000	0.0000	0.5000	0.02772 (14)	
S1	0.31842 (5)	0.50038 (6)	0.18505 (5)	0.03230 (18)	
O1	0.03394 (9)	0.0836 (3)	0.40310 (11)	0.0485 (4)	
O2	0.30985 (11)	0.3259 (3)	0.12698 (14)	0.0544 (5)	
O3	0.39387 (14)	0.4851 (2)	0.27876 (16)	0.0462 (5)	
O4	0.32299 (12)	0.6768 (3)	0.13379 (15)	0.0582 (5)	
O5	0.51977 (12)	−0.2553 (3)	0.41016 (16)	0.0738 (7)	
H5A	0.4803	−0.3193	0.3698	0.089*	
H5B	0.5698	−0.2876	0.3976	0.089*	
O6	0.51991 (12)	0.2033 (3)	0.38506 (14)	0.0657 (6)	
H6A	0.4802	0.2889	0.3531	0.079*	
H6B	0.5701	0.2527	0.3817	0.079*	
N1	0.34204 (14)	−0.00860 (19)	0.41343 (14)	0.0296 (4)	
N2	0.12317 (10)	−0.1123 (3)	0.52234 (10)	0.0370 (4)	
H2A	0.0802	−0.1148	0.5484	0.044*	
H2B	0.1766	−0.1754	0.5469	0.044*	
C1	0.27511 (17)	−0.0072 (2)	0.45320 (16)	0.0279 (5)	
H1	0.2932	−0.0044	0.5180	0.033*	
C2	0.18103 (17)	−0.0100 (2)	0.39791 (17)	0.0277 (5)	
C3	0.15424 (17)	−0.0111 (2)	0.29767 (16)	0.0316 (5)	
H3	0.0888	−0.0066	0.2600	0.038*	
C4	0.22217 (18)	−0.0186 (3)	0.25694 (16)	0.0334 (5)	
H4	0.2057	−0.0249	0.1923	0.040*	
C5	0.31554 (17)	−0.0164 (2)	0.31683 (16)	0.0320 (5)	
H5	0.3645	−0.0202	0.2919	0.038*	
C6	0.10639 (16)	−0.0082 (2)	0.44191 (16)	0.0302 (5)	
C7	0.21328 (17)	0.5140 (2)	0.20881 (19)	0.0303 (5)	
C8	0.21293 (18)	0.4744 (3)	0.30073 (19)	0.0348 (5)	
H8	0.2707	0.4455	0.3494	0.042*	
C9	0.12884 (18)	0.4791 (3)	0.31652 (19)	0.0349 (5)	
H9	0.1271	0.4538	0.3756	0.042*	
C10	0.04503 (16)	0.5228 (2)	0.24163 (18)	0.0311 (5)	
C11	0.04791 (14)	0.5662 (3)	0.15037 (15)	0.0353 (4)	
H11	−0.0095	0.5976	0.1017	0.042*	
C12	0.13142 (14)	0.5621 (3)	0.13407 (14)	0.0342 (4)	
H12	0.1338	0.5904	0.0754	0.041*	

### Atomic displacement parameters (Å^2^)

**Table:**

	*U*^11^	*U*^22^	*U*^33^	*U*^12^	*U*^13^	*U*^23^
Cd1	0.01281 (17)	0.03481 (19)	0.03689 (19)	−0.00051 (5)	0.01054 (11)	−0.00146 (6)
S1	0.0174 (3)	0.0352 (3)	0.0500 (4)	−0.00152 (14)	0.0190 (3)	−0.00351 (17)
O1	0.0210 (7)	0.0697 (11)	0.0582 (9)	0.0136 (8)	0.0181 (6)	0.0197 (9)
O2	0.0275 (8)	0.0627 (11)	0.0810 (11)	−0.0076 (8)	0.0290 (8)	−0.0316 (10)
O3	0.0196 (9)	0.0565 (11)	0.0619 (12)	−0.0001 (5)	0.0136 (8)	−0.0056 (6)
O4	0.0369 (9)	0.0594 (11)	0.0942 (13)	0.0054 (8)	0.0427 (9)	0.0243 (10)
O5	0.0318 (8)	0.0794 (13)	0.1183 (16)	−0.0128 (9)	0.0366 (10)	−0.0596 (13)
O6	0.0289 (8)	0.0791 (13)	0.0921 (13)	0.0042 (8)	0.0254 (8)	0.0459 (11)
N1	0.0152 (9)	0.0393 (10)	0.0338 (9)	−0.0004 (5)	0.0083 (7)	−0.0008 (6)
N2	0.0186 (7)	0.0542 (11)	0.0399 (9)	0.0035 (7)	0.0125 (6)	0.0069 (8)
C1	0.0161 (11)	0.0381 (12)	0.0295 (10)	−0.0004 (6)	0.0082 (8)	0.0001 (6)
C2	0.0189 (11)	0.0288 (10)	0.0353 (12)	0.0000 (6)	0.0095 (9)	−0.0004 (6)
C3	0.0184 (11)	0.0377 (12)	0.0340 (12)	0.0001 (6)	0.0036 (9)	0.0009 (7)
C4	0.0283 (12)	0.0421 (12)	0.0277 (10)	−0.0005 (7)	0.0075 (9)	−0.0006 (7)
C5	0.0228 (11)	0.0398 (11)	0.0362 (11)	0.0007 (7)	0.0139 (9)	−0.0008 (7)
C6	0.0154 (10)	0.0387 (12)	0.0352 (11)	−0.0019 (6)	0.0074 (8)	−0.0013 (7)
C7	0.0183 (11)	0.0288 (10)	0.0499 (13)	−0.0005 (6)	0.0197 (10)	−0.0014 (7)
C8	0.0212 (10)	0.0388 (10)	0.0483 (13)	0.0048 (7)	0.0172 (9)	0.0074 (8)
C9	0.0254 (11)	0.0388 (11)	0.0469 (12)	0.0054 (7)	0.0209 (10)	0.0098 (8)
C10	0.0200 (11)	0.0287 (9)	0.0508 (12)	0.0002 (7)	0.0202 (9)	−0.0007 (8)
C11	0.0195 (9)	0.0427 (11)	0.0454 (11)	0.0000 (9)	0.0139 (8)	−0.0004 (10)
C12	0.0237 (9)	0.0419 (10)	0.0412 (10)	−0.0029 (9)	0.0169 (8)	−0.0012 (10)

### Geometric parameters (Å, °)

**Table:**

Cd1—N1^i^	2.230 (2)	C1—C2	1.341 (3)
Cd1—N1	2.230 (2)	C1—H1	0.9300
Cd1—O5^i^	2.316 (2)	C2—C3	1.439 (3)
Cd1—O5	2.316 (2)	C2—C6	1.481 (3)
Cd1—O6^i^	2.3479 (19)	C3—C4	1.357 (4)
Cd1—O6	2.3479 (19)	C3—H3	0.9300
S1—O4	1.463 (2)	C4—C5	1.353 (3)
S1—O3	1.470 (2)	C4—H4	0.9300
S1—O2	1.4742 (19)	C5—H5	0.9300
S1—C7	1.717 (2)	C7—C12	1.373 (3)
O1—C6	1.200 (3)	C7—C8	1.434 (4)
O5—H5A	0.8108	C8—C9	1.346 (3)
O5—H5B	0.8551	C8—H8	0.9300
O6—H6A	0.8517	C9—C10	1.384 (4)
O6—H6B	0.8328	C9—H9	0.9300
N1—C1	1.332 (3)	C10—C10^ii^	1.439 (4)
N1—C5	1.387 (3)	C10—C11	1.443 (3)
N2—C6	1.368 (3)	C11—C12	1.341 (3)
N2—H2A	0.8600	C11—H11	0.9300
N2—H2B	0.8600	C12—H12	0.9300
			
N1^i^—Cd1—N1	180.00 (6)	C2—C1—H1	120.7
N1^i^—Cd1—O5^i^	87.37 (7)	C1—C2—C3	119.9 (2)
N1—Cd1—O5^i^	92.63 (7)	C1—C2—C6	118.8 (2)
N1^i^—Cd1—O5	92.63 (6)	C3—C2—C6	121.2 (2)
N1—Cd1—O5	87.37 (7)	C4—C3—C2	121.5 (2)
O5^i^—Cd1—O5	180.0	C4—C3—H3	119.2
N1^i^—Cd1—O6^i^	87.44 (7)	C2—C3—H3	119.2
N1—Cd1—O6^i^	92.56 (7)	C5—C4—C3	115.4 (2)
O5^i^—Cd1—O6^i^	86.23 (10)	C5—C4—H4	122.3
O5—Cd1—O6^i^	93.77 (10)	C3—C4—H4	122.3
N1^i^—Cd1—O6	92.56 (7)	C4—C5—N1	123.5 (2)
N1—Cd1—O6	87.44 (7)	C4—C5—H5	118.3
O5^i^—Cd1—O6	93.77 (10)	N1—C5—H5	118.3
O5—Cd1—O6	86.23 (10)	O1—C6—N2	124.3 (2)
O6^i^—Cd1—O6	180.0	O1—C6—C2	117.0 (2)
O4—S1—O3	114.71 (11)	N2—C6—C2	118.65 (18)
O4—S1—O2	111.55 (16)	C12—C7—C8	123.5 (2)
O3—S1—O2	113.62 (11)	C12—C7—S1	115.34 (19)
O4—S1—C7	106.64 (9)	C8—C7—S1	121.17 (18)
O3—S1—C7	102.88 (13)	C9—C8—C7	119.9 (2)
O2—S1—C7	106.44 (9)	C9—C8—H8	120.1
Cd1—O5—H5A	131.1	C7—C8—H8	120.1
Cd1—O5—H5B	129.3	C8—C9—C10	117.6 (2)
H5A—O5—H5B	97.4	C8—C9—H9	121.2
Cd1—O6—H6A	126.9	C10—C9—H9	121.2
Cd1—O6—H6B	130.0	C9—C10—C10^ii^	117.4 (3)
H6A—O6—H6B	97.1	C9—C10—C11	121.3 (2)
C1—N1—C5	120.96 (19)	C10^ii^—C10—C11	121.4 (3)
C1—N1—Cd1	121.05 (16)	C12—C11—C10	121.50 (19)
C5—N1—Cd1	117.99 (15)	C12—C11—H11	119.3
C6—N2—H2A	120.0	C10—C11—H11	119.3
C6—N2—H2B	120.0	C11—C12—C7	116.2 (2)
H2A—N2—H2B	120.0	C11—C12—H12	121.9
N1—C1—C2	118.7 (2)	C7—C12—H12	121.9
N1—C1—H1	120.7		

Symmetry codes: (i) −*x*+1, −*y*, −*z*+1; (ii) −*x*, *y*, −*z*+1/2.

### Hydrogen-bond geometry (Å, °)

**Table:**

*D*—H···*A*	*D*—H	H···*A*	*D*···*A*	*D*—H···*A*
O5—H5A···O3^iii^	0.81	2.03	2.832 (3)	171
O5—H5B···O4^iv^	0.86	1.83	2.676 (2)	172
O6—H6A···O3	0.85	1.93	2.777 (3)	178
O6—H6B···O2^v^	0.83	1.89	2.716 (3)	171
N2—H2A···O1^vi^	0.86	2.08	2.932 (2)	171
N2—H2B···O2^vii^	0.86	2.17	3.025 (3)	172

Symmetry codes: (iii) *x*, *y*−1, *z*; (iv) −*x*+1, *y*−1, −*z*+1/2; (v) −*x*+1, *y*, −*z*+1/2; (vi) −*x*, −*y*, −*z*+1; (vii) *x*, −*y*, *z*+1/2.
